# 5-{4′-[(5-Benzyl-2*H*-tetra­zol-2-yl)meth­yl]biphenyl-2-yl}-1*H*-tetra­zole monohydrate

**DOI:** 10.1107/S1600536813009963

**Published:** 2013-04-17

**Authors:** Gangadhar Y. Meti, S. Jeyaseelan, R. R. Kamble, Atakuri Dorababu, H. C. Devarajegowda

**Affiliations:** a Department of Studies in Chemistry, Karnataka University, Dharwad 580 003, Karnataka, India; b Department of Physics, St. Philomena’s College, Mysore 570 006, Karnataka, India; c Department of Physics, Yuvaraja’s College (Constituent College), University of Mysore, Mysore 570 005, Karnataka, India

## Abstract

In the title compound, C_22_H_18_N_8_·H_2_O, the dihedral angle between the tetra­zole rings is 69.58 (1)° while the terminal phenyl ring makes dihedral angles of 26.98 (8) and 39.75 (8)° with the other benzene rings. The rings of the biphenyl unit subtend a dihedral angle of 55.23 (8)°. In the crystal, the solvent water mol­ecule is linked to the main mol­ecule *via* an N—H⋯O hydrogen bond. In addition, C—H⋯N and O—H⋯N hydrogen bonds link the components into chains along [010]. The crystal structure also features C—H⋯π and π–π inter­actions, with centroid–centroid distances of 3.6556 (9) and 3.826 (1) Å.

## Related literature

For general background to biphenyl derivatives, see: Li *et al.* (2011 ▶); Tomori *et al.* (2000 ▶). For the synthesis and biological activity of tetra­zole derivatives, see: Kamble *et al.* (2011 ▶); Rao & Babu (2011 ▶). For biological properties of tetra­zole-derivatized biphenyl moieties, see: Zhang *et al.* (2008 ▶); Wang *et al.* (2010 ▶); Reddy *et al.* (2007 ▶). For related structures, see: Zhang *et al.* (2004 ▶). For the extinction correction, see: Larson (1970 ▶).
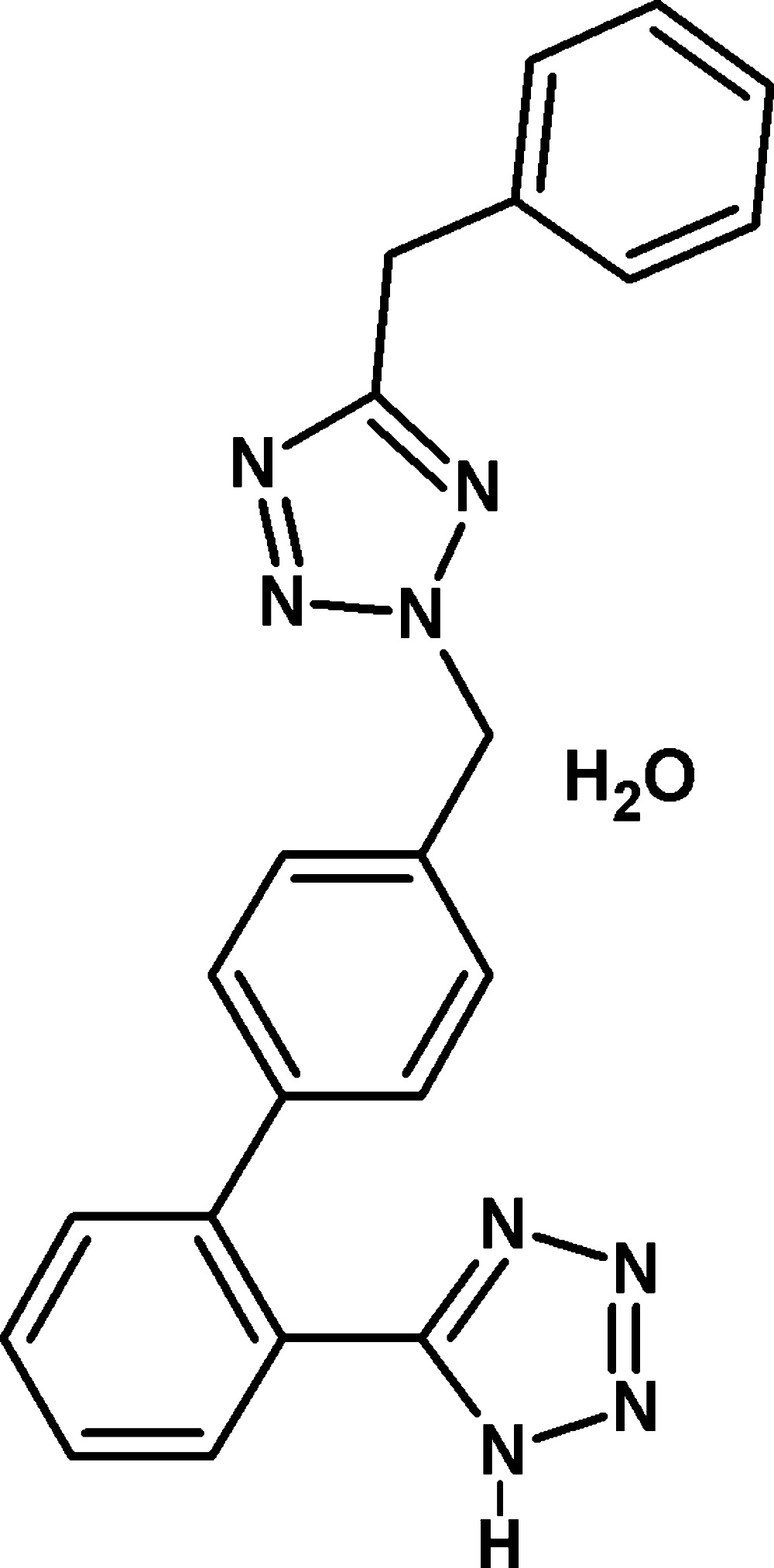


## Experimental

### 

#### Crystal data

C_22_H_18_N_8_·H_2_O
*M* *_r_* = 412.45
Monoclinic, 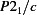

*a* = 14.7659 (4) Å
*b* = 7.6507 (3) Å
*c* = 18.2922 (5) Å
β = 97.153 (2)°
*V* = 2050.38 (11) Å^3^
*Z* = 4
Mo *K*α radiation
μ = 0.09 mm^−1^
*T* = 293 K
0.24 × 0.20 × 0.12 mm

#### Data collection

Bruker SMART CCD area-detector diffractometer
Absorption correction: ψ scan (*SADABS*; Sheldrick, 2007 ▶) *T*_min_ = 0.770, *T*_max_ = 1.000
18220 measured reflections
3923 independent reflections
3469 reflections with *I* > 2σ(*I*)
*R*_int_ = 0.026

#### Refinement

*R*[*F* ^2^ > 2σ(*F* ^2^)] = 0.048
*wR*(*F* ^2^) = 0.107
*S* = 1.00
3881 reflections
281 parameters
H-atom parameters constrained
Δρ_max_ = 0.49 e Å^−3^
Δρ_min_ = −0.41 e Å^−3^

### 

Data collection: *SMART* (Bruker, 2001 ▶); cell refinement: *SAINT* (Bruker, 2001 ▶); data reduction: *SAINT*; program(s) used to solve structure: *SIR92* (Altomare *et al.*, 1994 ▶); program(s) used to refine structure: *CRYSTALS* (Betteridge *et al.*, 2003 ▶); molecular graphics: *ORTEP-3* (Farrugia, 2012 ▶); software used to prepare material for publication: *CAMERON* (Watkin *et al.*, 1996 ▶).

## Supplementary Material

Crystal structure: contains datablock(s) I, global. DOI: 10.1107/S1600536813009963/hg5301sup1.cif

Structure factors: contains datablock(s) I. DOI: 10.1107/S1600536813009963/hg5301Isup2.hkl

Supplementary material file. DOI: 10.1107/S1600536813009963/hg5301Isup3.cml

Additional supplementary materials:  crystallographic information; 3D view; checkCIF report

## Figures and Tables

**Table 1:** Hydrogen-bond geometry (Å, °) *Cg*1, *Cg*3, *Cg*4 and *Cg*5 are the centroids of the C6/N2–N5 tetra­zole ring, the C8–C13 benzene ring, the C15–C20 benzene ring and the C21/C22/C28–C31 benzene ring, respectively.

*D*—H⋯*A*	*D*—H	H⋯*A*	*D*⋯*A*	*D*—H⋯*A*
C7—H71⋯N25^i^	0.99	2.59	3.514 (2)	156
N24—H241⋯O1^ii^	0.92	1.79	2.703 (2)	173
O1—H11⋯N27	0.83	2.35	2.950 (2)	130
C9—H91⋯*Cg*4^iii^	0.96	2.85	3.418 (1)	119
C12—H121⋯*Cg*1^i^	0.94	2.82	3.602 (1)	141
C14—H142⋯*Cg*3^iv^	0.96	2.72	3.676 (1)	171
C29—H291⋯*Cg*5^v^	0.96	2.80	3.682 (2)	153
C31—H311⋯*Cg*3^vi^	0.95	2.96	3.582 (1)	125

Symmetry codes: (i) 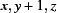; (ii) 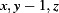; (iii) 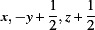; (iv) 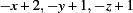; (v) 
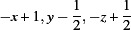
; (vi) 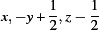.

**Fig. 1.**
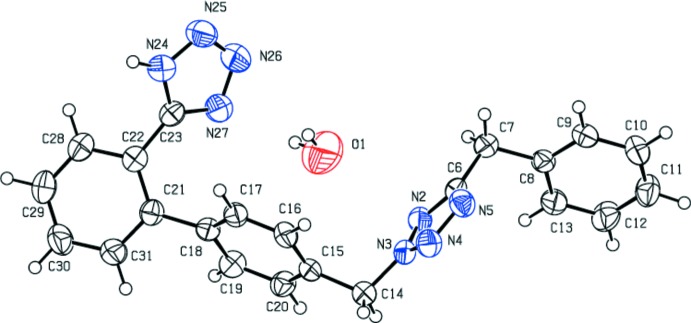
The molecular structure of the title compound. Displacement ellipsoids are drawn at the 50% probability level. Hydrogen atoms are shown as spheres of arbitrary radius.

**Fig. 2.**
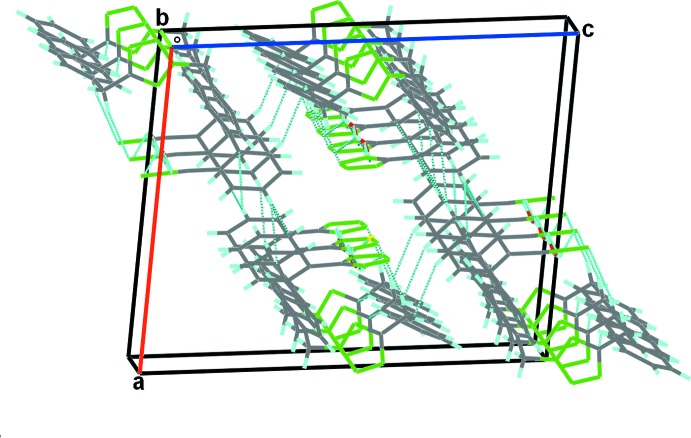
Crystal packing for the title compound with hydrogen bonds drawn as dashed lines.

## supplementary crystallographic information

### Comment

Biphenyl and tetrazole and derivatives are present in many of the bioactive heterocyclic compounds which are of wide interest because of their diverse pharmaceutical and clinical applications (Li *et al.*, 2011; Tomori *et al.*, 2000). As the tetrazole moiety functions as a carboxylic acid biostere that imparts the greater metabolic stability and increased absorption relative to the carboxylic acid. Tetrazole linked biphenyl moiety is the building block of all the Antihypertensive saratans (Rao & Babu, 2011; Reddy *et al.*, 2007; Wang *et al.*, 2010; Zhang *et al.*, 2008).

The asymmetric unit of 5-{4'-[(5-Benzyl-2*H*-tetrazol-2-yl) methyl] biphenyl -2-yl}-1*H*-tetrazole is shown in Fig. 1. the dihedral angle between the tetrazole (C6/N2/N3–N5, C23/C24/N25–N27) rings is 69.58 (1)° while the terminal phenyl ring (C8–C13) makes dihedral angles of 26.98 (8)° and 39.75 (8)° with the other benzene rings (C15–C20 & C21/C22/C28/C29–C31) and also dihedral angle for biphenyl (C15–C20 & C21\C22\C28\···C31 is 55.23 (8)°. In the crystal, the solvent water molecule is linked to the main molecule via N27—H12···O1 and O1—H11···N27 hydrogen bonds. In addition, there are intermolecular C7—H71···N25 hydrogen. The structure contains π-π, with a centroid–centroid (C_g_(1), C6/N2/N3–N5) and (C_g_(2), C23/24/N25–N27) distance of 3.6556 (9) Å and 3.826 (1) A° respectively and also C—H···π interactions (Table 1). In the structure, all bond lengths and angles are within normal ranges (Kamble *et al.*, 2011; Zhang *et al.*, 2004). The crystal packing shows stack the molecules along the *b* axis (Fig. 2).

### Experimental

Prepared using 4'-[(5-benzyl-2*H*-tetrazol-2-yl) methyl] biphenyl-2-carbonitrile according the method reported by Kamble *et al.* (2011). (mp. 393 K). Spectral data IR (KBr) cm^-1^ 3421 (N—H), 3125, 2985 (C—H of CH_2_), 1616 (C=C, str). ^1^H NMR (CDCl_3_): 7.3–8.00 (13 H, m Ar—H) 5.7 (2*H*, s, biphenyl CH_2_) 4.2 (2*H*, s, benzyl CH_2_). MS (m/*z*, 70 eV): 394 (*M*+), 382, 318, 235, 205,192, 178 (base peak),165, 91 and 77.

### Refinement

The H atoms were all located in a difference map, but those attached to carbon atoms were repositioned geometrically, with N–H = 0.91 Å, O–H = 0.83 Å and 0.96 Å, C—H = 0.93–0.98 Å for aromatic H and C—H = 0.97–0.99 Å for methylene H and refined using a riding model with *U*_iso_(H) = 1.2*U*_eq_(C) for aromatic and methylene H.

### Crystal data

**Table:**

C_22_H_18_N_8_·H_2_O	*F*(000) = 864
*M**_r_* = 412.45	*D*_x_ = 1.336 Mg m^−^^3^
Monoclinic, *P*2_1_/*c*	Melting point: 393 K
Hall symbol: -P 2ybc	Mo *K*α radiation, λ = 0.71073 Å
*a* = 14.7659 (4) Å	Cell parameters from 3923 reflections
*b* = 7.6507 (3) Å	θ = 1.4–25.9°
*c* = 18.2922 (5) Å	µ = 0.09 mm^−^^1^
β = 97.153 (2)°	*T* = 293 K
*V* = 2050.38 (11) Å^3^	Plate, colourless
*Z* = 4	0.24 × 0.20 × 0.12 mm

### Data collection

**Table:**

Bruker SMART CCD area-detector diffractometer	3923 independent reflections
Radiation source: fine-focus sealed tube	3469 reflections with *I* > 2σ(*I*)
Graphite monochromator	*R*_int_ = 0.026
ω/2θ scans	θ_max_ = 25.9°, θ_min_ = 1.4°
Absorption correction: ψ scan (*SADABS*; Sheldrick, 2007)	*h* = −18→18
*T*_min_ = 0.770, *T*_max_ = 1.000	*k* = −9→4
18220 measured reflections	*l* = −22→22

### Refinement

**Table:**

Refinement on *F*^2^	Hydrogen site location: difference Fourier map
Least-squares matrix: full	H-atom parameters constrained
*R*[*F*^2^ > 2σ(*F*^2^)] = 0.048	Method, part 1, Chebychev polynomial, (Watkin, 1994, *P*rince, 1982) [*w*eight] = 1.0/[A_0_*T_0_(x) + A_1_*T_1_(x) ··· + A_n-1_]*T_n-1_(x)] where A_i_ are the Chebychev coefficients listed belo*w* and x = *F* /*F*max Method = Robust Weighting (*P*rince, 1982) W = [*w*eight] * [1-(delta*F*/6*sigma*F*)^2^]^2^ A_i_ are: 2.08 2.65 0.641
*wR*(*F*^2^) = 0.107	(Δ/σ)_max_ = 0.0003916
*S* = 1.00	Δρ_max_ = 0.49 e Å^−^^3^
3881 reflections	Δρ_min_ = −0.41 e Å^−^^3^
281 parameters	Extinction correction: Larson (1970), eq. 22
0 restraints	Extinction coefficient: 194 (8)
Primary atom site location: structure-invariant direct methods	

### Special details

**Table:**

Refinement. Refinement of *F*^2^ against ALL reflections. The weighted *R*-factor *wR* and goodness of fit *S* are based on *F*^2^, conventional *R*-factors *R* are based on *F*, with *F* set to zero for negative *F*^2^. The threshold expression of *F*^2^ > 2σ(*F*^2^) is used only for calculating *R*-factors(gt) *etc*. and is not relevant to the choice of reflections for refinement. *R*-factors based on *F*^2^ are statistically about twice as large as those based on *F*, and *R*- factors based on ALL data will be even larger.

### Fractional atomic coordinates and isotropic or equivalent isotropic displacement parameters (Å^2^)

**Table:**

	*x*	*y*	*z*	*U*_iso_*/*U*_eq_	
O1	0.6409 (2)	0.1472 (2)	0.47303 (14)	0.1327	
N2	0.83657 (9)	0.38128 (17)	0.49755 (7)	0.0418	
N3	0.90644 (9)	0.31880 (16)	0.46640 (7)	0.0393	
N4	0.98138 (9)	0.29937 (19)	0.51215 (7)	0.0458	
N5	0.96150 (10)	0.35174 (19)	0.57699 (7)	0.0467	
C6	0.87317 (10)	0.40044 (19)	0.56678 (8)	0.0376	
C7	0.82354 (12)	0.4669 (2)	0.62728 (9)	0.0450	
C8	0.84982 (10)	0.6537 (2)	0.64806 (8)	0.0379	
C9	0.89345 (11)	0.6936 (2)	0.71693 (8)	0.0405	
C10	0.91827 (12)	0.8634 (2)	0.73543 (10)	0.0497	
C11	0.89843 (14)	0.9950 (2)	0.68486 (11)	0.0559	
C12	0.85406 (15)	0.9565 (2)	0.61566 (11)	0.0583	
C13	0.82998 (13)	0.7868 (2)	0.59720 (9)	0.0490	
C14	0.90005 (12)	0.2733 (2)	0.38826 (9)	0.0469	
C15	0.83532 (10)	0.1230 (2)	0.36911 (8)	0.0376	
C16	0.85021 (11)	−0.0387 (2)	0.40347 (8)	0.0413	
C17	0.79254 (11)	−0.1773 (2)	0.38405 (8)	0.0396	
C18	0.71895 (10)	−0.1599 (2)	0.32939 (8)	0.0377	
C19	0.70374 (11)	0.0023 (2)	0.29551 (9)	0.0441	
C20	0.76118 (11)	0.1420 (2)	0.31558 (9)	0.0435	
C21	0.66114 (10)	−0.3118 (2)	0.30243 (8)	0.0396	
C22	0.61489 (10)	−0.4190 (2)	0.34797 (8)	0.0393	
C23	0.61203 (10)	−0.3818 (2)	0.42664 (9)	0.0419	
N24	0.61342 (10)	−0.5040 (2)	0.47864 (8)	0.0503	
N25	0.60308 (12)	−0.4293 (3)	0.54318 (8)	0.0616	
N26	0.59523 (12)	−0.2645 (3)	0.53016 (9)	0.0658	
N27	0.60071 (11)	−0.2297 (2)	0.45804 (9)	0.0574	
C28	0.56465 (11)	−0.5620 (2)	0.31777 (10)	0.0479	
C29	0.55904 (12)	−0.5987 (3)	0.24391 (10)	0.0559	
C30	0.60313 (13)	−0.4927 (3)	0.19858 (10)	0.0575	
C31	0.65331 (12)	−0.3514 (2)	0.2277 (1)	0.0513	
H72	0.8360	0.3888	0.6694	0.0557*	
H71	0.7576	0.4619	0.6101	0.0561*	
H91	0.9083	0.6017	0.7535	0.0474*	
H101	0.9499	0.8872	0.7832	0.0609*	
H111	0.9198	1.1134	0.6966	0.0685*	
H121	0.8397	1.0480	0.5800	0.0720*	
H131	0.7977	0.7624	0.5483	0.0586*	
H142	0.9617	0.2406	0.3806	0.0582*	
H141	0.8769	0.3783	0.3599	0.0583*	
H161	0.9003	−0.0516	0.4409	0.0493*	
H171	0.8033	−0.2867	0.4087	0.0477*	
H191	0.6537	0.0202	0.2569	0.0536*	
H201	0.7493	0.2551	0.2919	0.0532*	
H281	0.5325	−0.6371	0.3493	0.0572*	
H291	0.5220	−0.6962	0.2232	0.0682*	
H301	0.6007	−0.5195	0.1476	0.0699*	
H311	0.6853	−0.2767	0.1966	0.0623*	
H12	0.5816	0.1027	0.4540	0.1934*	
H241	0.6242	−0.6206	0.4728	0.0639*	
H11	0.6563	0.0508	0.4913	0.1953*	

### Atomic displacement parameters (Å^2^)

**Table:**

	*U*^11^	*U*^22^	*U*^33^	*U*^12^	*U*^13^	*U*^23^
O1	0.203 (3)	0.0529 (11)	0.150 (2)	0.0060 (13)	0.0539 (19)	0.0055 (11)
N2	0.0411 (7)	0.0413 (7)	0.0432 (7)	−0.0038 (6)	0.0065 (5)	−0.0056 (6)
N3	0.0433 (7)	0.0357 (7)	0.0398 (7)	−0.0059 (5)	0.0082 (5)	−0.0056 (5)
N4	0.0450 (7)	0.0446 (8)	0.0475 (8)	0.0014 (6)	0.0041 (6)	−0.0028 (6)
N5	0.0513 (8)	0.0472 (8)	0.0403 (7)	0.0009 (6)	0.0000 (6)	−0.0014 (6)
C6	0.0474 (8)	0.0267 (7)	0.0391 (8)	−0.0051 (6)	0.0066 (6)	0.0003 (6)
C7	0.0564 (9)	0.0375 (8)	0.0431 (8)	−0.0063 (7)	0.0147 (7)	−0.0016 (7)
C8	0.0441 (8)	0.0344 (8)	0.0368 (7)	0.0008 (6)	0.0118 (6)	−0.0013 (6)
C9	0.0450 (8)	0.0420 (9)	0.0353 (7)	0.0063 (7)	0.0081 (6)	0.0020 (6)
C10	0.0564 (10)	0.0497 (10)	0.0419 (8)	0.0007 (8)	0.0013 (7)	−0.0093 (7)
C11	0.0668 (11)	0.0369 (9)	0.0628 (11)	−0.0026 (8)	0.0030 (9)	−0.0073 (8)
C12	0.0781 (13)	0.0379 (9)	0.0565 (10)	−0.0010 (9)	−0.0011 (9)	0.0108 (8)
C13	0.0627 (10)	0.0459 (9)	0.0369 (8)	−0.0045 (8)	0.0007 (7)	0.0036 (7)
C14	0.0559 (9)	0.0470 (9)	0.0396 (8)	−0.0124 (8)	0.0128 (7)	−0.0063 (7)
C15	0.0427 (8)	0.0384 (8)	0.0335 (7)	−0.0038 (6)	0.0116 (6)	−0.0059 (6)
C16	0.0439 (8)	0.0441 (9)	0.0346 (7)	−0.0004 (7)	0.0006 (6)	−0.0040 (6)
C17	0.0463 (8)	0.0362 (8)	0.0361 (7)	0.0005 (6)	0.0039 (6)	0.0005 (6)
C18	0.0382 (7)	0.0384 (8)	0.0374 (7)	−0.0011 (6)	0.0084 (6)	−0.0013 (6)
C19	0.0398 (8)	0.0453 (9)	0.0458 (9)	0.0023 (7)	−0.0002 (6)	0.0038 (7)
C20	0.0489 (9)	0.0352 (8)	0.0467 (8)	0.0012 (7)	0.0074 (7)	0.0033 (7)
C21	0.0350 (7)	0.0399 (8)	0.0433 (8)	−0.0007 (6)	0.0027 (6)	−0.0014 (7)
C22	0.0347 (7)	0.0385 (8)	0.0442 (8)	0.0028 (6)	0.0037 (6)	0.0013 (7)
C23	0.0368 (7)	0.0419 (9)	0.0473 (9)	−0.0043 (6)	0.0067 (6)	0.0016 (7)
N24	0.0527 (8)	0.0508 (8)	0.0479 (8)	−0.0012 (7)	0.0082 (6)	0.0028 (6)
N25	0.0634 (10)	0.0743 (12)	0.0490 (9)	−0.0070 (8)	0.0143 (7)	−0.0005 (8)
N26	0.0736 (11)	0.0714 (12)	0.0564 (9)	−0.0080 (9)	0.0236 (8)	−0.0117 (8)
N27	0.0702 (10)	0.0481 (9)	0.0574 (9)	−0.0025 (7)	0.0211 (8)	−0.0061 (7)
C28	0.0438 (8)	0.0432 (9)	0.0557 (10)	−0.0061 (7)	0.0030 (7)	0.0012 (7)
C29	0.0502 (10)	0.0518 (10)	0.0629 (11)	−0.0108 (8)	−0.0038 (8)	−0.0100 (9)
C30	0.0583 (10)	0.0660 (12)	0.0460 (9)	−0.0093 (9)	−0.0017 (8)	−0.0125 (9)
C31	0.0519 (9)	0.0587 (11)	0.0434 (8)	−0.0105 (8)	0.0067 (7)	−0.0026 (8)

### Geometric parameters (Å, º)

**Table:**

O1—H12	0.963	C15—C20	1.383 (2)
O1—H11	0.830	C16—C17	1.379 (2)
N2—N3	1.3281 (18)	C16—H161	0.947
N2—C6	1.3218 (19)	C17—C18	1.388 (2)
N3—N4	1.3101 (19)	C17—H171	0.954
N3—C14	1.4626 (19)	C18—C19	1.393 (2)
N4—N5	1.3191 (19)	C18—C21	1.489 (2)
N5—C6	1.347 (2)	C19—C20	1.385 (2)
C6—C7	1.491 (2)	C19—H191	0.966
C7—C8	1.517 (2)	C20—H201	0.974
C7—H72	0.975	C21—C22	1.405 (2)
C7—H71	0.985	C21—C31	1.390 (2)
C8—C9	1.376 (2)	C22—C23	1.473 (2)
C8—C13	1.386 (2)	C22—C28	1.396 (2)
C9—C10	1.381 (2)	C23—N24	1.332 (2)
C9—H91	0.977	C23—N27	1.317 (2)
C10—C11	1.374 (3)	N24—N25	1.337 (2)
C10—H101	0.955	N24—H241	0.915
C11—C12	1.382 (3)	N25—N26	1.286 (3)
C11—H111	0.975	N26—N27	1.358 (2)
C12—C13	1.377 (3)	C28—C29	1.372 (3)
C12—H121	0.962	C28—H281	0.978
C13—H131	0.978	C29—C30	1.380 (3)
C14—C15	1.508 (2)	C29—H291	0.973
C14—H142	0.971	C30—C31	1.380 (3)
C14—H141	0.994	C30—H301	0.952
C15—C16	1.392 (2)	C31—H311	0.970
			
H12—O1—H11	91.3	C15—C16—C17	120.61 (14)
N3—N2—C6	101.78 (12)	C15—C16—H161	118.9
N2—N3—N4	113.97 (12)	C17—C16—H161	120.5
N2—N3—C14	123.09 (13)	C16—C17—C18	120.98 (14)
N4—N3—C14	122.94 (13)	C16—C17—H171	119.5
N3—N4—N5	105.97 (12)	C18—C17—H171	119.5
N4—N5—C6	106.23 (13)	C17—C18—C19	118.31 (14)
N5—C6—N2	112.06 (14)	C17—C18—C21	121.92 (14)
N5—C6—C7	123.27 (14)	C19—C18—C21	119.60 (13)
N2—C6—C7	124.67 (14)	C18—C19—C20	120.66 (14)
C6—C7—C8	111.93 (13)	C18—C19—H191	121.2
C6—C7—H72	108.4	C20—C19—H191	118.2
C8—C7—H72	111.3	C19—C20—C15	120.74 (14)
C6—C7—H71	107.9	C19—C20—H201	119.8
C8—C7—H71	109.2	C15—C20—H201	119.4
H72—C7—H71	108.0	C18—C21—C22	124.06 (14)
C7—C8—C9	120.94 (14)	C18—C21—C31	118.03 (14)
C7—C8—C13	119.87 (14)	C22—C21—C31	117.91 (14)
C9—C8—C13	119.19 (15)	C21—C22—C23	122.78 (14)
C8—C9—C10	120.84 (15)	C21—C22—C28	119.75 (15)
C8—C9—H91	120.5	C23—C22—C28	117.37 (14)
C10—C9—H91	118.6	C22—C23—N24	124.20 (15)
C9—C10—C11	119.77 (16)	C22—C23—N27	128.14 (15)
C9—C10—H101	119.2	N24—C23—N27	107.47 (15)
C11—C10—H101	121.0	C23—N24—N25	109.70 (16)
C10—C11—C12	119.85 (17)	C23—N24—H241	126.0
C10—C11—H111	119.8	N25—N24—H241	124.1
C12—C11—H111	120.2	N24—N25—N26	105.85 (15)
C11—C12—C13	120.23 (17)	N25—N26—N27	110.93 (15)
C11—C12—H121	120.3	N26—N27—C23	106.06 (15)
C13—C12—H121	119.4	C22—C28—C29	120.93 (16)
C8—C13—C12	120.11 (16)	C22—C28—H281	120.0
C8—C13—H131	120.8	C29—C28—H281	119.0
C12—C13—H131	119.1	C28—C29—C30	119.74 (16)
N3—C14—C15	111.74 (12)	C28—C29—H291	120.1
N3—C14—H142	104.8	C30—C29—H291	120.1
C15—C14—H142	110.4	C29—C30—C31	119.95 (17)
N3—C14—H141	107.1	C29—C30—H301	120.0
C15—C14—H141	109.2	C31—C30—H301	120.0
H142—C14—H141	113.6	C21—C31—C30	121.71 (17)
C14—C15—C16	120.83 (14)	C21—C31—H311	117.4
C14—C15—C20	120.47 (14)	C30—C31—H311	120.8
C16—C15—C20	118.68 (14)		

### Hydrogen-bond geometry (Å, º)

Cg1, Cg3, Cg4 and Cg5 are the centroids of the C6/N2–N5 tetrazole ring, the C8–C13 benzene ring, the C15–C20 benzene ring and the C21/C22/C28–C31 benzene ring, respectively.

**Table:**

*D*—H···*A*	*D*—H	H···*A*	*D*···*A*	*D*—H···*A*
C7—H71···N25^i^	0.99	2.59	3.514 (2)	156
N24—H241···O1^ii^	0.92	1.79	2.703 (2)	173
O1—H11···N27	0.83	2.35	2.950 (2)	130
C9—H91···*Cg*4^iii^	0.96	2.85	3.418 (1)	119
C12—H121···*Cg*1^i^	0.94	2.82	3.602 (1)	141
C14—H142···*Cg*3^iv^	0.96	2.72	3.676 (1)	171
C29—H291···*Cg*5^v^	0.96	2.80	3.682 (2)	153
C31—H311···*Cg*3^vi^	0.95	2.96	3.582 (1)	125

Symmetry codes: (i) *x*, *y*+1, *z*; (ii) *x*, *y*−1, *z*; (iii) *x*, −*y*+1/2, *z*+1/2; (iv) −*x*+2, −*y*+1, −*z*+1; (v) −*x*+1, *y*−1/2, −*z*+1/2; (vi) *x*, −*y*+1/2, *z*−1/2.

## References

[bb1] Altomare, A., Cascarano, G., Giacovazzo, C., Guagliardi, A., Burla, M. C., Polidori, G. & Camalli, M. (1994). *J. Appl. Cryst.* **27**, 435.

[bb2] Betteridge, P. W., Carruthers, J. R., Cooper, R. I., Prout, K. & Watkin, D. J. (2003). *J. Appl. Cryst.* **36**, 1487.

[bb3] Bruker (2001). *SMART* and *SAINT.* Bruker AXS Inc., Madison, Wisconsin, USA.

[bb4] Farrugia, L. J. (2012). *J. Appl. Cryst.* **45**, 849–854.

[bb5] Kamble, R. R., Biradar, D. B., Meti, G. Y., Taj, T., Gireesh, T., Khazi, I. M., Vaidynathan, S. T., Mohandoss, R., Sridhar, B. & Parthasarathi, V. (2011). *J. Chem. Sci.* **123**, 393–401.

[bb6] Larson, A. C. (1970). Crystallographic Computing, edited by F. R. Ahmed, S. R. Hall & C. P. Huber, pp. 291–294. Copenhagen: Munksgaard.

[bb7] Li, W., Xu, Z., Sun, P., Jiang, X. & Fang, M. (2011). *Org. Lett.* **13**, 1286–1289. 10.1021/ol103075n 21338092

[bb8] Rao, S. N. & Babu, K. S. (2011). *Org. Commun.* **4**, 105–111.

[bb9] Reddy, K. S., Srinivasan, N., Reddy, C. R., Kolla, N., Anjaneyulu, Y., Venkatraman, S., Bhattacharya, A. & Mathad, V. T. (2007). *Org. Process Res. Dev.* **11**, 81–85.

[bb10] Sheldrick, G. M. (2007). *SADABS* Bruker AXS Inc., Madison, Wisconsin, USA.

[bb11] Tomori, H., Fox, J. M. & Buchwald, S. L. (2000). *J. Org. Chem.* **65**, 5334–5341. 10.1021/jo000691h 10993363

[bb12] Wang, P., Zheng, G., Wang, Y., Wang, X., Li, Y. & Xiang, W. (2010). *Tetrahedron*, **66**, 5402–5406.

[bb14] Watkin, D. J., Prout, C. K. & Pearce, L. J. (1996). *CAMERON* Chemical Crystallography Laboratory, Oxford, England.

[bb15] Zhang, H., Yang, B., Zheng, Y., Yang, G., Ye, L., Ma, Y., Chen, X., Cheng, G. & Liu, S. (2004). *J. Phys. Chem.* **108**, 9571–9573.

[bb16] Zhang, C. X., Zheng, G. J., Bi, F. Q. & Li, Y. L. (2008). *Chin. Chem. Lett.* **19**, 759–761.

